# Illustrations of the Comparative Anatomy of the Nervous System

**Published:** 1839-07

**Authors:** 


### Art. XI.

*Illustrations of the Comparative Anatomy of the Nervous System.* By Joseph Swan. Parts III. and IV.—*London*, 1837—1839. 4to, pp. 53 to 106. Pl. 14 to 25.

On a former occasion we expressed our opinion of the utility of this work, and the present numbers have justified the approbation we then bestowed upon it. We are perfectly satisfied that it will tend still further to advance the high reputation which Mr. Swan has already acquired, as a practical anatomist of the nervous system. It is much to be hoped that the example set in this work, of graphically illustrating the structure and distribution of the nervous system in the different classes of animals, will soon be followed by illustrations of the other organs of the body. Works of this description, as we before remarked, are much wanted, and would prove highly useful to the student in comparative anatomy, by serving as guides to direct him in his practical examinations of the internal organs in different classes of animals. Nearly all the great discoveries in the structure and functions of the different parts of the human body have derived their origin from, or have been confirmed and rendered practically useful through, the aid of comparative anatomical examinations of similar structures in other animals. It was by examining the structure of the vascular system in the lower animals, and by comparing the phenomena which occurred in them in the living state with similar phenomena in the human body, that Harvey succeeded in demonstrating the circulation of the blood; and it is only by the aid of similar means,—the performing of experiments upon the lower animals, but which necessarily requires a previous knowledge of the structure of the parts in the animals operated upon,—that the functions of many parts in the human body can be fully elucidated. In our own time, the splendid discovery of Sir Charles Bell of the distinct functions of the roots of the spinal nerves was obtained by means of comparative anatomy, being first demonstrated by Bell, Magendie, and others, upon the lower animals; and this important addition to our knowledge, "the second great discovery in physiology," has recently been more fully confirmed by Müller, in his experiments upon the cold-blooded vertebrata. These facts alone are sufficient to show the utility of works such as the one now before us, and we strongly hope that what Mr. Swan is now doing for the comparative anatomy of the nervous system, will be done by him, or some other able anatomist, for that of the other important parts of the body.

We have before noticed the beauty of the engravings in Mr. Swan's work, as admirable specimens of art, but we cannot help observing, that we could have wished that he had executed the drawings for them himself, as a few inaccuracies and blemishes observable in some of the plates, and which detract a little from their value, would then, probably, have been avoided. We are quite certain that anatomical drawings by an anatomist, even when but roughly executed, are much to be preferred, and will always convey a more vivid idea of the object, than those which are made by a professed artist unacquainted with anatomical peculiarities. One of the faults to which we particularly allude in these drawings is the stiffness with which some of the objects are represented. They remind us more of carved wooden structures than of the free and pliable vessels of a living animal body. An instance of this is seen in plate XV., in the delineation of the heart of the turtle, with its vessels, the pulmonary artery and right aorta. Bold and vigorous delineations by an anatomist would have conveyed a much more correct notion of these parts than the finished drawing before us. We cannot better explain our preference, than by requesting our readers to compare with these the graphic delineations of some of the arteries of the human body, in one of the earlier publications of Sir Charles Bell, some of which were both drawn and etched by Sir Charles himself, with a vigour and truth to nature hardly to be surpassed.

With these remarks upon the execution of the plates, we now turn with pleasure to an examination of their contents. Plate XVII. is the one which interests us most. It has been stated by some anatomists that the spinal nerves of the python, and other ophidians, have but one root derived from the spinal cord; but Mr. Swan has clearly shown, in his delineation of a portion of the cord of the boa-constrictor, that two roots certainly do exist, notwithstanding what has been asserted to the contrary with regard to the python. This, indeed, we should have expected would be the fact, if the opinion respecting the difference of function in the two roots of the nerves in the higher animals were correct, as it is now proved to be by Müller's experiments upon the roots of the nerves in frogs. Mr. Swan also notices an equally interesting fact with respect to the cord itself, which, in the boa, is continued to the end of the tail, and "has cineritious matter within it similar to that in mammalia." He also remarks that "each anterior bundle (or root of the spinal nerves) is rather larger than the posterior." (p. 64.) This difference in the size of the roots of the spinal nerves is an important characteristic, inasmuch as it seems to indicate the preponderance of the motor over the sensitive functions, in this class of animals; and hence, from the circumstance that in the human subject and the higher vertebrata the posterior roots are the largest, while the function of sensation appears to be developed to the greatest amount, leads us to infer that the preponderance of either function in the spinal nerves of all animals corresponds with their relative size.

In some of his dissections of birds, which we had looked for with much interest, Mr. Swan has been particularly happy; especially in his tracing of the sympathetic system, to which he seems to have paid most attention. We could have wished that he had confined himself simply to anatomical details, since, in the few instances in which he has ventured to theorize upon the uses of the structures he describes, he has not been so fortunate in his conclusions as in his dissections of the parts themselves. Thus he states (p. 103), that "in the pelican the greatest part of the nerves supplying the upper and lower jaw, and the pharynx or great bag, are covered with a black membrane, which is provided on account of the thinness of this extensive part, and for preventing any privation of the influence of the nerves, and a consequent retardation of the circulation of the blood, from exposure to a low temperature." We certainly do not perceive the conclusiveness of this assumption, since if this be really the use of the membrane, we might fairly expect to find a similar membrane enveloping the nerves in all parts of like thinness, and exposed to an equally low temperature, as in the web of the feet of the pelican, and in that of the swan and other water-birds, but such does not appear to have been found by Mr. Swan, in his dissections of these birds. While, on the other hand, he states (at p. 77), that in the crocodile "the nervous system is almost entirely covered by a black membrane." Again, in his attempts to generalize on the utility of certain parts of the body, he is not more fortunate; as when he states (p. 103), that "parts covered with feathers generally receive spinal nerves, and seldom branches of the fifth; hairs, in the human body and animals, exist in parts supplied by both, but the longer hairs and wool in particular, in those furnished by the spinal; there are exceptions to all these statements; it is, therefore, most probable that the structure of the skin, independently of the nerves, is a principal cause of the difference." Here he attempts to indicate a law with reference to the influence which particular nerves exert over the production of wool, hair, or feathers; forgetting, in the first place, that, according to the views now generally received, the fifth nerves and the spinal nerves are precisely analogous; and next, that the appendages of the skin, whether in the form of wool, hair, or feathers, are only modifications of the same structure, and are dependent, not upon the particular nerves distributed to the part in which they are produced, but upon the skin itself, for their peculiar formation. It is in such anatomical details, then, as are exhibited in Plates XXI, XIV, and XXV, that Mr. Swan appears to most advantage; and in his summary of the differences which exist in the structure of corresponding parts of different animals, as in the following observations on the brain.

"The brain of birds differs from that of many of the amphibia, in the form and comparatively large size of the hemispheres. In the greater dimensions of the anterior lobes, in proportion to the olfactory nerves; in the very thin parietes of the posterior part of the lateral ventricles; in the shape and magnitude of the striated bodies; in the larger size and different situation of the optic thalami; in the flattened summit, but greater circumference of the optic lobe; and in the shape of the cavity, which corresponds with their external form. In the external shape and appearance of the cerebellum; in its having convolutions; in the thickness of the parietes of its small cavity, which communicates with the other ventricles. In the absence of longitudinal eminences extending towards the calamus scriptorius, and forming the ventricular cord; in the greater thickness of the oblong medulla. It agrees in the presence of the anterior, posterior, and soft commissures; in crura extending to the oblong medulla, and in the similar arrangement and origin of the nerves. It differs from that of many of the mammalia, in the want of convolutions and the corpus callosum, or great commissure. In the great thinness of the posterior parietes of the lateral ventricles; in the radiated form of the septum, and the different construction of the floor of this cavity; in its being continuous with the sides of the ventricles, and not separate, like the fornix. In the hollow optic lobes, instead of the quadrigeminal bodies. It agrees in having small lateral lobes, similar to the lobules attached to the sides of the cerebellum in the monkey and some other animals, and also in their being placed in a hollow, partly surrounded by a semicircular canal, which in man, and some animals, is occupied by part of the petrous portion of bone. It differs in the continuation of the third ventricle into the cerebellum, as well as into the calamus scriptorius; in the want of the annular tubercle, although there is a considerable enlargement of the oblong medulla at this part." (p. 100.)

We are surprised that in this summary Mr. Swan has not noticed the similarity which exists between the brain of birds and marsupial animals, in the absence of the great commissure, as pointed out some time since by Mr. Owen, in the Philosophical Transactions. But he has noticed one peculiarity in the muscular system which we do not remember to have before met with. He states that "in the pinion very distinct little muscles are observed for giving motions to the quills, and thus form a variety of the cutaneous muscle." (p. 98.)

In conclusion, we beg again to recommend Mr. Swan's work to the student in comparative anatomy; at the same time, we hope that the forthcoming numbers will appear a little more quickly, since only four numbers have as yet been published, the first of which appeared in 1835, and the third in 1837, while the fourth has but recently made its appearance.

---

